# Four Year Clinical and Cost Effectiveness of Vaginal Pessary Self‐Management Versus Clinic‐Based Care for Pelvic Organ Prolapse (TOPSY): Long Term Follow‐Up of a Randomised Controlled Superiority Trial

**DOI:** 10.1111/1471-0528.18333

**Published:** 2025-08-20

**Authors:** Carol Bugge, Rohna Kearney, Catherine Best, Kirsteen Goodman, Sarkis Manoukian, Lynn Melone, Melanie Dembinsky, Helen Mason, Andrew Elders, Margaret Graham, Wael Agur, Suzanne Breeman, Jane Culverhouse, Lucy Dwyer, Mark Forrest, Karen Guerrero, Christine Hemming, Aethele Khunda, Angela Kucher, Doreen McClurg, John Norrie, Ranee Thakar, Suzanne Hagen

**Affiliations:** ^1^ School of Health and Life Sciences/Research Centre for Health Glasgow Caledonian University Glasgow UK; ^2^ The Warrell Unit, Saint Mary's Hospital, Manchester University NHS Foundation Trust Manchester Academic Health Science Centre Manchester UK; ^3^ Division of Developmental Biology and Medicine, School of Medical Sciences University of Manchester Manchester UK; ^4^ Faculty of Health Sciences and Sport University of Stirling Stirling UK; ^5^ Centre for Healthcare Randomised Trials (CHaRT), Institute of Applied Health Sciences University of Aberdeen Aberdeen UK; ^6^ Yunus Centre for Social Business and Health Glasgow Caledonian University Glasgow UK; ^7^ Statistics Department Phastar London UK; ^8^ PPI Representative Glasgow UK; ^9^ NHS Ayrshire & Arran Kilmarnock UK; ^10^ University of Glasgow Glasgow UK; ^11^ Division of Nursing, Midwifery and Social Work, School of Health Sciences University of Manchester Manchester UK; ^12^ NHS Greater Glasgow and Clyde Glasgow UK; ^13^ Aberdeen Royal Infirmary Aberdeen UK; ^14^ South Tees Hospitals NHS Foundation Trust James Cook University Hospital Middlesbrough UK; ^15^ Retired, c/o Glasgow Caledonian University Glasgow Scotland UK; ^16^ Centre for Public Health Queen's University Belfast Belfast UK; ^17^ Croydon University Hospital Croydon UK; ^18^ St George's University of London London UK

**Keywords:** long‐term follow‐up, pelvic organ prolapse, pessary, randomised controlled trial, self‐management

## Abstract

**Objective:**

To compare long‐term clinical and cost‐effectiveness of pessary self‐management (SM) with clinic‐based care (CBC) for pelvic floor‐specific quality of life (QoL).

**Design:**

Four‐year questionnaire follow‐up of trial participants.

**Setting:**

UK pessary clinics.

**Sample:**

Responders at 4 years aged ≥ 18 years at recruitment, using a pessary (except Shelf, Gellhorn or Cube) which had been retained ≥ 2 weeks. Exclusions: limited manual dexterity; cognitive deficit; pregnancy; requiring non‐English SM teaching.

**Methods:**

SM group received a 30‐min teaching session; information leaflet; 2‐week follow‐up call; and telephone support. CBC group received routine appointments. Allocation was by remote web‐based application, minimised on age, user type (new/existing) and centre with no blinding. Participants were invited to opt into a 4‐year follow‐up. The primary analysis was intention to treat.

**Outcome Measures:**

The primary outcomes were pelvic floor‐specific QoL (PFIQ‐7) and incremental net monetary benefit (INB) 4 years post‐randomisation. Secondary outcomes included complications and prolapse symptoms.

**Results:**

Of 340 women randomised, 186 (55%) responded at 4 years (86/169 [51%] SM, 100/171 [58%] CBC). There was no statistically significant group difference in PFIQ‐7 at 4 years (mean SM 32.9 vs. CBC 31.4, adjusted mean difference [AMD] SM‐CBC 4.86, 95% CI −6.41 to 16.12). There was a statistically non‐significant lower percentage of pessary complications for SM (SM 17.7% vs. CBC 22.0%, AMD 3.01 CI −0.58 to 6.61). At 4‐years, SM was cost‐effective (INB £2240). There was one potentially related serious adverse event (SM group).

**Conclusions:**

Pessary self‐management is an effective and cost‐effective long‐term option for women with prolapse.

**Trial Registration:**

ISRCTN number: 62510577 (https://doi.org/10.1186/ISRCTN62510577)

## 1 Introduction

Worldwide, 5%–10% of women experience symptomatic pelvic organ prolapse [1]. Treatment options include lifestyle changes, pelvic floor muscle training, vaginal pessary and surgery [1]. Pessaries are a commonly used, first‐line treatment globally [2, 3].

There is limited long‐term data on pessary use [4, 5]. Observational studies have reported long‐term pessary continuation rates from 45% to 86% at ~5 years [4, 6, 7, 8, 9, 10, 11], the variance possibly due to different care delivery models, global locations and pessary types.

Two main pessary care delivery models are used: clinic‐based care (CBC) (clinic appointments at routine intervals for pessary removal, cleaning and renewal by a healthcare professional) and self‐management (SM) (woman removes, cleans and renews her pessary herself) [5, 12]. Practice varies, with some countries providing more and others less SM [8, 13, 14].

Only one randomised controlled trial (RCT) compares SM to CBC, reporting at 18 months post‐randomisation that SM did not improve or worsen women's quality of life (QoL) led to fewer complications, and was cost‐effective compared to CBC [15, 16]. This paper reports on the long‐term follow‐up of these trial participants.

Two recent reviews summarise mainly observational study evidence about pessary SM [14, 17]. A scoping review including 23 publications concluded that SM offers women benefits without increasing risk, but some women do not feel able to self‐manage [14]. A systematic review exploring SM adherence identified seven publications, concluding that SM led to high continuation rates with low conversion to surgery [17]. This review reported no significant difference in pessary continuation rates between SM and CBC; however, others reported SM as a positive predictor of pessary continuation [6, 9, 13]. Other reported advantages of SM include fewer complications and greater convenience and comfort [8, 13, 18], these studies often rely on retrospective chart review and short‐term follow‐up. The current study evaluated the clinical and cost‐effectiveness of pessary SM, compared to CBC, on the pelvic floor‐specific QoL of women with prolapse, 4 years post‐randomisation.

## 2 Methods

### 2.1 Study Design

Long‐term questionnaire follow‐up of participants from a parallel‐group multicentre RCT assessing the superiority of pessary SM over CBC for women with prolapse. Individuals were randomised in a 1:1 allocation ratio. The original trial follow‐up completed at 18 months post‐randomisation [15, 16, 19, 20, 21].

### 2.2 Participants

Participants were women and people (hereafter women) with prolapse who took part in the original trial and opted into a 4‐year follow‐up from 21 UK National Health Service (NHS) pessary care settings. New pessary users (pessary use ≤ 3 months) or existing users (pessary use > 3 months) were originally approached by clinic staff. Women were eligible if they were ≥ 18 years, using a pessary of any type or material (except Shelf, Gellhorn or Cube) and had successfully retained the pessary for at least 2 weeks. They were ineligible if pregnant, experienced limited manual dexterity that prevented SM, had a cognitive deficit that prevented informed consent or SM (as judged by the treating healthcare professional), or had insufficient understanding of the English language (SM information only provided in English).

### 2.3 Randomisation and Masking

Remote allocation was undertaken by centre staff via a web‐based system with minimisation on age (< 65/≥ 65 years), pessary user type (new/existing) and centre. Participants, intervention deliverers, researchers and the statistician were not blinded to group allocation.

### 2.4 Procedures

Centre staff were trained in intervention delivery during a site visit and given an intervention manual. Women randomised to SM received: a 30‐min SM support session with a trained healthcare professional where they were taught and practised pessary insertion and removal; a SM information leaflet; a follow‐up phone call 2 weeks after the support session to check they had been able to successfully self‐manage and troubleshoot any problems; and a local telephone number to access pessary team support if required [22]. Women randomised to CBC returned to clinic to have their pessary removed, cleaned, and replaced/renewed by a healthcare professional at intervals as per usual practice at their centre [22]. Most centres had return appointments every 6 months [16].

Eighteen months after randomisation, the participant's clinical care was returned to the centre. Women could then practice SM or receive CBC dependent on what was routinely offered and their personal preference. Follow‐up appointments for women in both groups were arranged in line with usual practice.

### 2.5 Adherence

Adherence to trial group protocol was assessed using data from participant‐completed questionnaires.

### 2.6 Outcomes

Four‐year clinical effectiveness was assessed for pelvic floor‐specific QoL [19], measured using the Pelvic Floor Impact Questionnaire‐7 (PFIQ‐7) (primary outcome measure), a validated, participant‐completed instrument with total score ranging from 0 to 300 (higher scores reflect worse pelvic floor‐specific QoL) [23]. The PFIQ‐7, one of a small selection of Patient Reported Outcome Measures for prolapse [24], was chosen as it was used for two large trials of pessary use at the outset of this study [25, 26]. For cost‐effectiveness, the incremental net monetary benefit (INB) at 4 years is presented to bring costs and benefits together in one value that can be used for decision‐making.

Secondary outcome measures at 4 years were the same as those used at 18 months [16]: the EuroQol EQ‐5D‐5L [27]; the Pelvic Floor Distress Inventory‐20 (PFDI‐20) [23]; the Prolapse Incontinence Sexual Questionnaire‐IUGA Revised version (PISQ‐IR) [28]; the Patient Global Impression of Improvement (PGI‐I) [29]; the General Self‐Efficacy Scale [30]; study‐specific pessary questionnaires (pessary complications, use and confidence); and uptake of additional prolapse treatment. At 4 years, the International Physical Activity Questionnaire for the Elderly (IPAQ‐E) was added (four items, scored using MET minutes [metabolic equivalent of a task] over 1 week [31]) to measure physical activities that are part of everyday life [32]. Resource‐use data (primary and secondary care use, for example, hospital or clinic appointments and prescribed drugs) were collected using a study‐specific Health Resource Use Questionnaire.

Outcome data were collected by participant‐completed questionnaire at 4 years; and all previous time‐points (paper or online completion).

Between 18 months and 4 years, centres were asked to report any adverse and serious adverse events (SAEs) and trial staff examined participant questionnaire returns and followed up any possible events with centres. The following common pessary‐related concerns were not considered adverse events: granulation of vaginal tissue, involuntary expulsion of pessary, vaginal smell, vaginal discharge, and bleeding during pessary change.

### 2.7 Statistics

In the original trial, a sample size of 330 women (165 per group) was set to allow detection of a 20‐point difference in PFIQ‐7 score at the 18‐month follow‐up (based on 90% power and assuming a standard deviation of 50, two‐sided alpha of 0.05, and 20% loss to follow‐up). Randomised women who opted into the 4‐year follow‐up were sent the questionnaire. Analysis of 4‐year data was carried out in accordance with a statistical analysis plan approved prior to data collection completion. For all primary outcome analyses, 95% confidence intervals were calculated and reported for the estimates, and model assumptions were checked using visual assessment of residual plots.

The main 4‐year analysis followed the intention‐to‐treat (ITT) principal: participants were included and analysed according to their randomised group. Any missing PFIQ‐7 responses were assumed ‘missing at random’. A longitudinal analysis of covariance [33], which adjusted for age group, pessary user type and baseline scores, was used to test for a difference between groups. The models included a random effect for centre and participant, with an independent correlation structure.

A pre‐specified analysis of PFIQ‐7 was conducted, reflecting the following definition of ‘on treatment’ at 4‐years: women who respond ‘yes’ to the question asking whether they are self‐managing and have received SM training versus those who respond ‘no’ or did not answer the SM question. A mixed‐effects regression model estimated the difference between ‘on treatment’ and ‘not on treatment’ groups, including baseline PFIQ‐7, age group and pessary user type as covariates.

The impact of missing primary clinical outcome data was assessed in two analyses: a complete case analysis, using only cases where 4‐year PFIQ‐7 was available (missing completely at random); and a pattern mixture model increasing and decreasing the imputed PFIQ‐7 values by the MCID of 20 points (missing not at random), while adjusting for baseline values. Additionally, a post hoc sensitivity analysis was performed including PFIQ‐7 data from all timepoints, which used various alternative reference values to replace missing values (missing not at random) (e.g., last mean carried forward) [30, 34].

Pre‐specified subgroup analyses of the primary clinical outcome were carried out for age (< 65/65+ years), pessary user type (new/existing), previous hysterectomy (yes/no) and use of any hormone therapy at baseline. A stricter threshold for significance of *p* < 0.01 was set for these analyses.

As the 18‐month finding was one of no difference between groups in PFIQ‐7, an equivalence test was performed on the 4‐year primary outcome, testing the null hypothesis that the trial groups were different by 20 points (the MCID).

Secondary 4‐year outcomes were analysed using ITT and ‘on treatment’ definitions. PFDI‐20 and proportion of pessary complications were analysed using longitudinal analysis of covariance adjusted for baseline values and minimisation covariates with random effects of participant and centre. PISQ‐IR, IPAQ‐E, general self‐efficacy scale and pessary confidence (problem‐solving, insertion, removal) were analysed using mixed effects regression adjusted for baseline value (where collected) and minimisation covariates with a centre random effect. PGI‐I was analysed using ordinal logistic regression adjusted for minimisation variables.

### 2.8 Economic Evaluation

Cost‐utility analysis (CUA) was conducted from a health sector payer (UK NHS) perspective. Quality adjusted life year (QALYs) were calculated for the SM and CBC groups using the EQ‐5D‐5L. EQ‐5D questionnaire responses were converted into utility values by mapping EQ‐5D‐5L responses into the EQ‐5D‐3L using an algorithm that controls for age and gender [35] and using the UK tariff [36]. Costs were attached to self‐reported health care resource use and the intervention resources using published unit costs [37, 38]. All costs are reported in British Pounds (GBP£) in 2019 and 2020 prices (deflated and discounted to the 2019/2020 prices so that costs are comparable across all time points). A discount rate of 3.5% was applied to all costs and outcomes over 1 year [39].

The incremental cost and incremental effectiveness in QALYs of SM compared to CBC were calculated along with the incremental cost effectiveness ratio (ICER) and INB [40]. The INB was calculated by translating both effectiveness and cost into a monetary valuation using a willingness to pay per QALY gained of £20 000 [39, 41]. Positive INBs imply that SM is the cost‐effective option; negative INBs suggest the opposite. In sensitivity analysis, ‘data‐extrapolation’ was employed in cases where there was a gap in QoL measurement (at a follow‐up). We extrapolated the value using information from adjacent follow‐ups to maximise usable data.

### 2.9 Patient and Public Involvement

Patient and Public Involvement (PPI) has been central since study inception, including intervention development [22]. One PPI member was a study co‐applicant, and two PPI members were part of the project management group and involved in all aspects of the study. All are authors on this manuscript; and one documented her experiences [42].

## 3 Results

### 3.1 Respondent Sample and Characteristics

**FIGURE 1:**
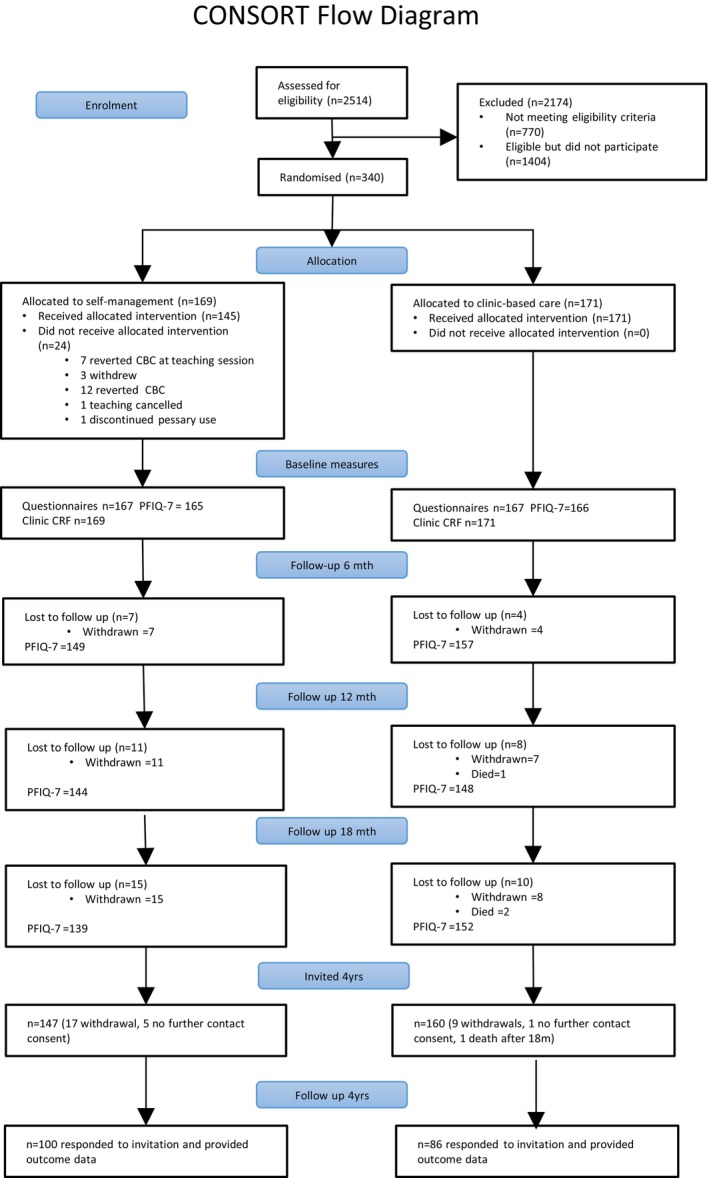
Consort diagram.

Originally, 2514 women were screened for eligibility (16th May 2018 to 7th February 2020) at 21 UK‐based centres (Figure 1), with 340 women randomised (169 SM; 171 CBC). The primary outcome, reported at 18 months, demonstrated no significant difference between groups in PFIQ‐7 (mean SM 32.3 vs. CBC 32.5, adjusted mean difference [AMD] SM‐CBC −0.03, 95% CI −9.32 to 9.25) [16]. At 4 years, 186 women (55% of the original 340 women) opted into the study and provided outcome data (51% [86/169] SM; 58% [100/171] CBC).

There did not seem to be any substantial difference in the participant characteristics of those who did (55%) and did not (45%) provide 4‐year follow‐up. Additionally, amongst the 55% responders, we obtained roughly equal numbers (86 vs. 100) and there was no strong evidence of meaningful differences in the participant characteristics between these randomised groups (Supporting Information: Tables A and B).

### 3.2 Primary Outcome

There were PFIQ‐7 data for all 186 responders at 4‐years. The ITT analysis showed no statistically significant difference between the groups in PFIQ‐7 score at 4‐years post‐randomisation (mean SM 32.9 [SD 56.6] vs. CBC mean 31.4 [SD 52.5], AMD 4.86, 95% CI −6.41 to 16.12, *p* = 0.398) (Table 1; Supporting Information: Figure A). Differences between groups, both adjusted and unadjusted, are lower than the MCID of 20 points. Pre‐specified sensitivity analyses of the PFIQ‐7 at 4 years showed no significant between‐group difference under different data assumptions, nor did an ‘on treatment’ analysis (Supporting Information: Figure B). Subgroup interactions for age group, pessary use, hormone therapy and hysterectomy at baseline were not found to be statistically significant (Supporting Information: Figure C). A test that the groups were different (by 20 points on the PFIQ‐7) was rejected (*t*[184] = 2.317 *p* = 0.0108), supporting the hypothesis of group equivalence (Supporting Information: Table C).

**TABLE 1:** Results of primary outcome measure (PFIQ‐7) intention to treat analysis by time point.

	Self‐management (SM)			Clinic‐based care (CBC)			Unadjusted mean difference (SM‐CBC) (95% CI)^a^	Adjusted mean difference (SM‐CBC) (95% CI)^b^	Effect size (adjusted estimate/pooled SD)
	*N*	Mean	SD	*N*	Mean	SD			
Baseline	165	32.5	49.6	166	31.7	48.0			
6 Months	149	22.7	36.7	157	29.4	47.7	−6.71 (−16.31 to 2.89)	−5.90 (−15.00 to 3.20)	0.138
12 Months	144	30.3	52.0	148	33.1	53.3	−2.78 (−14.90 to 9.35)	−3.45 (−12.71 to 5.82)	0.066
18 Months	139	32.3	50.9	152	32.5	47.8	−0.17 (−11.55 to 11.22)	−0.03 (−9.32 to 9.25)	0.001
4 Years	86	32.9	56.6	100	31.4	52.5	−1.46 (−17.25 to 14.33)	4.86 (−6.41 to 16.12)	0.090

*Note:* PFIQ‐7 range from 0 to 300 with higher scores indicating poorer quality of life.

Abbreviations: CBC, clinic‐based care; SM, self‐management.

^a^ Unadjusted analysis included no random effects or covariates.

^b^ Adjusted for age group, pessary user type (new vs. existing) and baseline PFIQ‐7 score and included random intercepts for participant and centre. Treatment effect sizes were estimated from the linear combination of the fixed effect solutions for the interaction between randomisation group (SM or CBC) and time‐point and the coefficient group in the mixed models.

### 3.3 Secondary Outcomes

At 4 years, participants reported on whether or not they experienced 15 pessary‐related complications, if applicable to them, in the previous 6 months (Table 2). Participants in the SM group reported a lower proportion of complications than those in the CBC group, but the between‐group difference was not statistically significant (SM 17.9% CBC 22.0%, AMD 3.01, 95% CI −0.58 to 6.61 *p* = 0.101). The three most common pessary‐related complications were urinary incontinence, vaginal discharge, and difficulty in bowel emptying.

**TABLE 2:** Self‐reported pessary complications at 4‐years.

	Self‐management (SM)			Clinic‐based care (CBC)			Total		
	*n*	*N*	Col %	*n*	*N*	Col %	*n*	*N*	Col %
Vaginal discharge	28	86	32.6	30	98	30.6	58	184	**31.5**
Vaginal smell	10	85	11.8	18	98	18.4	28	183	15.3
Vaginal pain	6	85	7.1	8	98	8.2	14	183	7.7
Urine infection	9	86	10.5	17	97	17.5	26	183	14.2
Urinary incontinence	44	85	51.8	53	97	54.6	97	182	**53.3**
Difficulty emptying bladder	12	85	14.1	27	98	27.6	39	183	21.3
Bowel incontinence	16	85	18.8	22	97	22.7	38	182	20.9
Difficulty emptying bowel	23	84	27.4	34	97	35.1	57	181	**31.5**
Unable to remove pessary	5	86	5.8	4	100	4.0	9	186	4.8
Difficulty removing pessary	14	85	16.5	9	98	9.2	23	183	12.6
Difficulty having sex	1	84	1.2	9	97	9.3	10	181	5.5
Pain during sex	1	83	1.2	8	98	8.2	9	181	5.0
Pessary fell out	10	84	11.9	12	98	12.2	22	181	12.2
Non‐menstrual bleeding	13	85	15.3	19	98	19.4	32	183	17.5
Problems other	8	79	10.1	2	92	2.2	10	171	5.8

	*N*	Mean (SD)	*N*	Mean (SD)	*N*	Mean (SD)
No. of complications reported as % of number relevant^a^	86	17.91 (15.66)	100	21.97 (17.14)	186	20.09 (16.55)

*Note:* Data are *n*/*N* (%) or mean (SD). Bold text highlights the three most common complications.

^a^ Percentage of complication types reported calculated for each participant; only 13 out of 15 categories applicable to both groups were included.

At 4 years, there were no significant between‐group differences for prolapse symptoms (PFDI‐20), sexual activity (PISQ‐IR), weekly physical activity (IPAQ‐E) or odds of being in a PGI‐I category indicating a more improved impression of pessary care (Table 3).

**TABLE 3:** Results of ITT analysis of other secondary outcomes at 4 years.

	Self‐management (SM)		Clinic‐based care (CBC)		Adjusted mean difference (SM‐CBC) between groups (95% CI)
	*N*	Mean (SD)	*N*	Mean (SD)	
PFDI‐20^a^	86	94.3 (55.7)	100	102.8 (53.5)	−5.07 (95% CI −15.91 to 5.76)
PISQ‐IR^b^	33	2.9 (1.2)	39	3.1 (1.0)	0.18 (95% CI −0.31 to 0.66)
IPAQ‐E^b^ (MET minutes)	70	3089.6 (5090.9)	83	4672.9 (6793.0)	−1583.28 (95% CI −3502.72 to 336.15)
General Self‐efficacy^b^	83	32.5 (4.7)	99	32.5 (4.8)	−0.19 (95% CI −1.30 to 0.92)
Confident manage pessary problems^b^	86	81.0 (22.9)	98	72.8 (27.2)	8.53 (95% CI 1.30–15.76)
Confident insert pessary^b^	86	82.3 (32.6)	99	55.9 (40.7)	26.56 (95% CI 16.00–37.12)
Confident remove pessary^b^	86	85.5 (29.5)	99	59.6 (39.7)	26.21 (95% CI 16.13–36.29)

	*N*	*n* (%)	*N*	*n* (%)	Odds ratio
PGI‐I, better^c^	85	29 (34.1%)	99	28 (28.3%)	1.01 (0.59–1.70)

Abbreviations: IPAQ‐E, International Physical Activity Questionnaire for the Elderly; PFDI‐20, Pelvic Floor Distress Inventory‐20; PGI‐I: Patient Global Impression of Improvement; PISQ‐IR, Prolapse Incontinence Sexual Questionnaire‐IUGA Revised version.

^a^ Longitudinal analysis of covariance adjusted for minimisation covariates with random effects of participant and centre.

^b^ Models not longitudinal—dependent variable outcome at 4 years adjusted for baseline value and minimisation covariates with random effect of centre.

^c^ Ordinal regression with adjustment for minimisation covariates.

There were no group differences in general self‐efficacy; however, self‐managing women were significantly more confident in their ability to manage pessary‐related problems, insert, and remove their pessary (Table 3).

Between 18 months and 4 years, 11 SM and 8 CBC women reported SAEs. Three participants had two SAEs, and one participant had five (related to gall bladder and breast cancer treatment). Two events were investigated for causality: one in the CBC group was an expected event as per protocol and the other (SM group) a possibly related event which linked to urinary and chest infections resulting in hospitalisation. All other events were unrelated to the intervention.

### 3.4 Pessary Use and Care Pathways at 4 Years

Pessary continuation rates were high with 93% (173/186) of responders having used a pessary within the last 6 months (92% [79/86] SM; 94% [94/100] CBC); and 89% (165/186) currently using a pessary (87.2% [75/86] SM; 90.0% [90/100] CBC). Of those randomised to the SM group and currently using a pessary, 80% (60/75) continued to self‐manage (i.e., 15 crossed over to CBC) and 39% (35/90) currently using a pessary of the original CBC group had crossed over to self‐manage by the 4‐year follow‐up (i.e., 55 stayed in the CBC group). Half (*n* = 43) of those from the original SM group and 45 (45%) of the CBC group reported having received SM training since the 18‐month follow‐up. Eight (4%) respondents had undergone surgery: 4 (2%) had undergone prolapse surgery (1 [1%] SM; 3 [3%] CBC); one woman (SM group) had undergone incontinence surgery; and 3 women (2 [2%] SM; 1 [1%] CBC) had a hysterectomy.

### 3.5 Cost‐Effectiveness

Data were available from 169 (78 SM, 91 CBC) participants for the CUA. The sample excluded 17 participants (8 SM, 9 CBC) who dropped out at baseline or had missing data in either EQ‐5D‐5L or resource questions at previous follow‐ups.

There were no group differences in EQ‐5D scores and visual analogue scales at any time point (Supporting Information: Table E). There was a significant difference in resource use from baseline to 18 months [15], between 18 months and 4 years, there was no statistically significant difference in resource use between the two groups (Supporting Information: Table F).

SM was less costly than CBC and was not less effective in terms of the number of QALYs gained from treatment (Table 4). The positive INB (£2240) demonstrates that SM is cost‐effective compared to CBC and means that the cost to derive the benefit from SM is less than the maximum amount that the decision‐maker would be willing to pay for this benefit. The probability SM is cost‐effective was more than 92% at a willing‐to‐pay threshold of £20 000 per QALY gained (Table 4; Supporting Information: Figure D).

**TABLE 4:** Distribution of incremental costs and effects associated with self‐management compared to clinic‐based care over the 4 years of the TOPSY study (intention to treat sample).

	*N* ^b^	Total cost (£GBP)	Total QALYs	Incremental cost (bootstrapped SE^a^)	Incremental QALYs (bootstrapped SE^a^)	ICER	INB (bootstrapped SE^a^)
Self‐management	78	£976.74	3.268	−239.06 (176.44)	0.100 (0.073)	Dominated	£2240.15 (£1530.25)
Clinic‐based care	91	£1215.80	3.168				
Probability of cost‐effectiveness at £20 000 WTP				92.97%			

^a^ Standard error based on 10 000 bootstrap resamples of incremental cost and effects.

^b^ Full completionsample used data where participants have responded to all questions fully without any gaps at follow ups. Reason for non‐inclusion was that some participants did not respond to questions at certain follow‐ups.

On treatment, analysis revealed a statistically significant difference in resource use between the two groups at 4 years. The probability of cost‐effectiveness for SM was 71%, which was lower than the primary analysis because the sample included participants who were self‐managing and were outliers in terms of their QoL (Supporting Information: Table G).

## 4 Discussion

### 4.1 Main Findings

There was no difference in long‐term pelvic floor‐specific QoL between those randomised to SM and those who received CBC. A high proportion of women in both groups continued to use a pessary 4 years after study enrolment, and conversion to surgery rates were low. There was no statistical between‐group difference in the proportion of complications experienced. The magnitude of difference was the same as at 18 months and in favour of the SM group. There was one potentially related SAE in the SM group over 4 years. SM women were more confident in their ability to insert and remove their pessary and to manage pessary‐related complications. SM is cost‐effective in the long term.

### 4.2 Strengths and Limitations

This is the only RCT comparing SM to CBC. As such, it provides robust evidence to inform clinical practice, and evidence of the safety of pessary SM which can reassure women considering pessary management for prolapse. Although the loss to follow up was 45%, Supporting Information: Table A demonstrates that the demographic and clinical characteristics of the responder and non‐responder groups, including co‐morbidities, were similar. Loss to follow‐up of participants at 4 years did result in a loss of statistical power, which may explain the lack of a statistical difference in complications between the SM and CBC groups.

Our participants predominately self‐reported their ethnicity as White. This was also the case in a recent survey in an ethnically diverse UK location (95.5% White background) [43]. While it is unclear why uptake is skewed towards those of White ethnicity, it does mean our findings have limited generalisability to those from diverse ethnic backgrounds.

### 4.3 Interpretation

In the long‐term, women who self‐managed had similar pelvic floor‐specific and general health‐related QoL to those who attended clinic for pessary care. This finding was robust as it held under different assumptions, across a range of sensitivity analyses, and a test of equivalence. This strengthens the finding from 18 months [16] and extends that knowledge into the long‐term. Up to 4 years there was one possibly‐related SAE. Although there are no other clinical trials comparing SM to CBC, three observational studies offer some comparison; two using retrospective chart review [6, 13] and one using quality improvement methodology [18]. None of these studies report on women's QoL, and only one has longer‐term follow‐up (3 years [6]), but all do suggest that there are few adverse events associated with SM. Available evidence would therefore suggest that SM is a viable and safe option for women who use a vaginal pessary for prolapse in the long‐term.

The SM group had a lower proportion of complications than those who undertook CBC; however, unlike 18‐month findings [16], the difference was not statistically significant. The body of evidence tends towards SM leading to fewer complications [13, 18, 44, 45]. One potential explanation is the confidence self‐managing women have to remove their pessary themselves, perhaps removing it more often. However, evidence from a study of 123 women suggests that lengthening the interval between pessary cleaning/change does not impact complications [46]. Other possible explanations include self‐managing women personalising the use of their pessary and having longer pessary‐free intervals than CBC women, and SM women having more confidence to manage pessary‐related problems themselves, thus addressing issues quickly.

The pessary continuation rate in our study at 4 years was 93%; higher than previously published figures of 45%–86% [4, 6, 7, 8, 9, 10, 11]. It is possible that the 4‐year follow‐up non‐responders did not respond because they had discontinued pessary use, and this may partly explain these higher continuation rates. It is also possible that COVID lockdown led to women continuing with a pessary due to a lack of other options.

Conversion to surgery (4%) was lower than the rates in other studies (12%–35%) [8, 10, 47, 48]. In the USA, Chen et al. [8] reported a statistical difference in surgery uptake favouring pessary SM (35.1% CBC; 19.3% SM). In our case, it may be that the long wait for prolapse surgery has meant that UK women have not been able to access surgery; a higher proportion would actually have converted to surgery than observed [49, 50].

The proposed mechanism of action of the SM intervention was that women who self‐managed would experience greater self‐efficacy and hence improved QoL. General self‐efficacy did not differ between the randomised groups, nor did QoL. However, women who were originally randomised to SM (ITT analysis) were more confident in their ability to remove and insert their pessary and to manage pessary‐related complications at 4 years. Given the extent of crossover to SM from CBC between 18 months and 4 years, the maintenance of this difference across time is perhaps surprising. A potential explanation may be that at 4 years, women self‐reported whether or not they received SM training; it is therefore possible that the training they reported was less comprehensive than that provided within the trial.

SM was a cost‐effective option when compared with CBC at 4 years for women who use a pessary for prolapse. This is consistent with previously published findings [15] with SM having similar QoL outcomes to CBC but using less resource. The difference was driven by lower resource use in the SM group over the first 18 months, with the difference narrowing over time. However, the ‘on treatment’ analysis identified that resource use was lower in the group who were actually self‐managing. Evidence from our study [15] and from elsewhere [8, 14, 15, 18] may provide an explanation, with SM leading to fewer clinic appointments, less contact with healthcare professionals in women's health more generally, and lower conversion to surgery [8]. In services where waiting lists are long, SM may be a helpful approach to open appointment slots and therefore decrease waiting time for appointments to secondary care and may prevent progression of symptoms [50].

## 5 Conclusion

Self‐management is a safe, effective and cost‐effective option for women using a vaginal pessary for prolapse. Clinical services should consider implementing robust self‐management protocols for women with the capacity to self‐manage. Future research should identify the best ways of implementing self‐management in different clinical contexts and ethnic minority groups.

## Author Contributions

Conception of the study: C.B. (lead), R.K. (co‐lead), H.M., A.E., M.G., W.A., S.B., L.D., M.F., K.G., C.H., A.Kh., D.McC., J.N., R.T., S.H. (co‐lead). Planning: C.B., R.K., K.Go., S.M., L.M., M.D., H.M., A.E., M.G., W.A., S.B., L.D., M.F., K.G., C.H., A.Kh., D.McC., J.N., R.T., S.H. Carrying out: All authors. Analysis: C.Be., S.H., C.B., S.M., H.M., R.K., M.D. Led manuscript writing: C.B. with S.H. and C.Be. All authors were involved in interpreting the data plus reading, commenting upon, and taking the decision to submit the manuscript for publication. All authors have approved the final submitted version.

## Ethics Statement

The trial received ethical approval from the West of Scotland Research Ethics Service, West of Scotland REC 3 (17/WS/0267) on 17th February 2018, with subsequent approval for the long‐term follow‐up received on 19th April 2022. Participants were provided with information about the 4 year follow up and asked to opt into the study by returning an expression of interest, with one reminder sent. Those who returned the expression of interest were sent a questionnaire 4 years after their initial randomisation.

## Conflicts of Interest

All authors were involved in the NIHR grant on which this manuscript is based (NIHR 16/82/01). Several authors are grant holders on additional grants from NIHR and/or charities. In addition: L.D. holds an NIHR fellowship (NIHR300519), is a member of the NIHR HTA secondary care prioritisation committee, has received payment from Mediplus Ltd for delivering training on pessary management, is a committee member of the UK pessary guidelines committee; M.G. is a PPI reviewer for NIHR Health Technology Assessment; R.K. has received consultancy payments from the British Standards Institute; A.Ku. is on the Advisory Panel as a PPI contributor for the NIHR study referenced NIHR151938; W.A. has received consulting fees and payment for testimony from Oaklaw Consultancy Ltd. for Medico‐legal Consultancy and has a financial/non‐financial interest associated with Medical Innovation System; R.T. is the President of the Royal College of Obstetrics and Gynaecology.

## Supporting information


**Data S1:** bjo18333‐sup‐0001‐DataS1.docx.

## Data Availability

The data that support the findings of this study are available on request from the corresponding author. The data are not publicly available due to privacy or ethical restrictions.
