# Supplementary material for: Four Year Clinical and Cost Effectiveness of Vaginal Pessary Self‐Management Versus Clinic‐Based Care for Pelvic Organ Prolapse (TOPSY): Long Term Follow‐Up of a Randomised Controlled Superiority Trial
Source: BJOG. 2025 Aug 20;132(12):1762–71. doi: 10.1111/1471-0528.18333 (PMC12501713; doi:10.1111/1471-0528.18333)
Supplement: Supplementary file 1 — Data S1: bjo18333‐sup‐0001‐DataS1.docx. [file BJO-132-1762-s001.docx]

**Supporting** **Table A: Comparison of responders at 4 years v non-responders at 4 years on baseline characteristics**

|  | **Not included 4 years** | | **Included 4 years** | | **Total** | |
| --- | --- | --- | --- | --- | --- | --- |
| ***Baseline characteristic*** | **n** | **Mean (SD)** | **n** | **Mean (SD)** | **n** | **Mean (SD)** |
| **Age** | 154 | 61.62 (13.01) | 186 | 65.44 (9.44) | 340 | 63.71 (11.34) |
| **PFIQ-7** | 148 | 37.63 (55.68) | 183 | 27.56 (41.91) | 331 | 32.06 (48.73) |
|  | | | | | | |
|  | **n** | **%** | **n** | **%** | **n** | **%** |
| **Education**  **No formal qualifications** | 19/120 | 15.8 | 18/153 | 11.8 | 37/273 | 13.6 |
| **Secondary/ Further Ed** | 49/120 | 40.8 | 61/153 | 39.9 | 110/273 | 40.3 |
| **Higher Ed** | 52/120 | 43.3 | 74/153 | 48.4 | 126/273 | 46.2 |
| **Employment**  **Full time** | 41/154 | 26.6 | 19/185 | 10.3 | 60/339 | 17.7 |
| **Part time** | 24/154 | 15.6 | 40/185 | 21.6 | 64/339 | 18.9 |
| **Student** | 4/154 | 2.6 | 0/185 | 0.0 | 4/339 | 1.2 |
| **At home** | 13/154 | 8.4 | 10/185 | 5.4 | 23/339 | 6.8 |
| **Seeking work** | 1/154 | 0.7 | 0/185 | 0.0 | 1/339 | 0.3 |
| **Other** | 71/154 | 46.1 | 116/185 | 62.7 | 187/339 | 55.2 |
| **Ethnicity**  **Any White** | 131/148 | 88.5 | 178/184 | 96.7 | 309/332 | 93.1 |
| **Any Asian** | 5/148 | 3.4 | 2/184 | 1.1 | 7/332 | 2.1 |
| **Any African** | 5/148 | 3.4 | 0/184 | 0.0 | 5/332 | 1.5 |
| **Any Caribbean** | 4/148 | 2.7 | 2/184 | 1.1 | 6/332 | 1.8 |
| **Mixed/ Other** | 2/148 | 1.4 | 2/184 | 1.1 | 4/332 | 1.2 |
| **Prefer not to say** | 1/148 | 0.7 | 0/184 | 0.0 | 1/332 | 0.3 |
|  | | | | | | |
| **Co-morbidities/ other treatments** | | | | | | |
|  | n/154 | % | n/186 | % | n/340 | % |
| **Any** **Hormone Therapy** | 42 | 27.3 | 67 | 36.0 | 109 | 32.1 |
| **Systemic HRT** | 7 | 4.5 | 6 | 3.2 | 13 | 3.8 |
| **Local oestrogen** | 37 | 24.0 | 61 | 32.8 | 98 | 28.8 |
| **Chronic Cough** | 9 | 5.8 | 12 | 6.5 | 21 | 6.2 |
| **Diabetes** | 4 | 2.6 | 9 | 4.8 | 13 | 3.8 |
| **Arthritis** | 43 | 27.9 | 45 | 24.2 | 88 | 25.9 |
| **Constipation** | 32 | 20.8 | 33 | 17.7 | 65 | 19.1 |
| **Recurrent UTIs** | 14 | 9.1 | 12 | 6.5 | 26 | 7.6 |
| **Vulvodynia** | 4 | 2.6 | 1 | 0.5 | 5 | 1.5 |
| **Hysterectomy** | 20 | 13.0 | 18 | 9.7 | 38 | 11.2 |
| **Pelvic Floor Surgery** | 16 | 10.4 | 23 | 12.4 | 39 | 11.5 |

**Supporting** **Table B: Baseline characteristics of 4-year responders by randomised group**

|  | **Self-Management (SM)** | | **Clinic-Based Care (CBC)** | | **Total** | |
| --- | --- | --- | --- | --- | --- | --- |
| ***Baseline characteristic*** | **n** | **Mean (SD)** | **n** | **Mean (SD)** | **n** | **Mean (SD)** |
| **Age** | 86 | 64.9 (10.1) | 100 | 65.9 (8.9) | 186 | 65.4 (9.4) |
| **PFIQ-7** | 85 | 25.6 (39.7) | 98 | 29.3 (43.8) | 183 | 27.6 (41.9) |
|  | | | | | | |
|  | **n** | **%** | **n** | **%** | **n** | **%** |
| **Education**  **No formal qualifications** | 8 | 11.3 | 10 | 12.2 | 18 | 11.8 |
| **Secondary/ Further Ed** | 29 | 40.9 | 32 | 39.0 | 61 | 39.9 |
| **Higher Ed** | 34 | 47.9 | 40 | 48.8 | 74 | 48.4 |
| **Employment**  **Full time** | 6 | 7.0 | 13 | 13.1 | 19 | 10.3 |
| **Part time** | 15 | 17.4 | 25 | 25.3 | 40 | 21.6 |
| **Student** | 0 | 0 | 0 | 0 | 0 | 0 |
| **At home** | 7 | 8.1 | 3 | 3.0 | 10 | 5.4 |
| **Seeking work** | 0 | 0 | 0 | 0 | 0 | 0 |
| **Other** | 58 | 67.4 | 58 | 58.6 | 116 | 62.7 |
| **Ethnicity**  **Any White** | 84 | 97.7 | 94 | 96.0 | 178 | 96.7 |
| **Any Asian** | 1 | 1.2 | 1 | 1.0 | 2 | 1.1 |
| **Any African** | 0 | 0 | 0 | 0 | 0 | 0 |
| **Any Caribbean** | 0 | 0 | 2 | 2.0 | 2 | 1.1 |
| **Mixed/ Other** | 1 | 1.2 | 1 | 1.0 | 2 | 1.1 |
| **Prefer not to say** | 0 | 0 | 0 | 0 | 0 | 0 |
|  | | | | | | |
| **Co-morbidities/ other treatments** | | | | | | |
|  | **Self-Management (SM)** | | **Clinic-Based Care (CBC)** | | **Total** | |
|  | n/86 | % | n/100 | % | n/186 | % |
| **Any Hormone Therapy** | 28 | 32.6 | 39 | 39.0 | 67 | 36.0 |
| **Systemic HRT** | 2 | 2.3 | 4 | 4.0 | 6 | 3.2 |
| **Local Oestrogen** | 26 | 30.2 | 35 | 35.0 | 61 | 32.8 |
| **Chronic Cough** | 7 | 8.1 | 5 | 5.0 | 12 | 6.5 |
| **Diabetes** | 4 | 4.7 | 5 | 5.0 | 9 | 4.8 |
| **Arthritis** | 22 | 25.6 | 23 | 23.0 | 45 | 24.2 |
| **Constipation** | 18 | 20.9 | 15 | 15.0 | 33 | 17.7 |
| **Recurrent UTIs** | 5 | 5.8 | 7 | 7.0 | 12 | 6.5 |
| **Vulvodynia** | 1 | 1.2 | 0 | 0.0 | 1 | 0.5 |
| **Hysterectomy** | 9 | 10.5 | 9 | 9.0 | 18 | 9.7 |
| **Pelvic Floor Surgery** | 12 | 14.0 | 11 | 11.0 | 23 | 12.4 |

**Supporting** **Table C: Test of equivalence for Primary Outcome (PFIQ-7)**

| Group | n | Mean | Std. err. | Std. dev. | 95% CI | |
| --- | --- | --- | --- | --- | --- | --- |
| SM | 86 | 32.85345 | 6.101901 | 56.58671 | 20.72124 | 44.98567 |
| CBC | 100 | 31.39683 | 5.249068 | 52.49068 | 20.98154 | 41.81212 |
| Δ-θ |  | 18.54337 | 8.003414 |  |  |  |
| θ+Δ |  | 21.45663 | 8.003414 |  |  |  |

θ = mean(PFIQ7TreatmentNo = 1) - mean(PFIQ7TreatmentNo = 2); Δ = 20.0 Δ expressed in same units as PFIQ7 df = 184; Ho: θ >= Δ: t1 = 2.317, t2 = 2.681. Ho1: Δ-θ <= 0 Ho2: θ+Δ <= 0 Ha1: Δ-θ > 0 Ha2: θ+Δ > 0 Pr(T > t1) = 0.0108 Pr(T > t2) = 0.0040

**Supporting** **Table D: Results of ‘on treatment’ analysis of other secondary outcomes at 4 years**

|  | On treatment | | Not on treatment | | Adjusted mean difference (on-not) between groups (95%CI) |
| --- | --- | --- | --- | --- | --- |
|  | N | Mean (SD) | N | Mean (SD) |  |
| PFDI-20 | 92 | 98.8 (59.3) | 70 | 97.7 (46.2) | 4.10 (95% CI -9.89 to 18.08) |
| PISQ-IR | 42 | 2.9 (1.2) | 21 | 3.2 (0.8) | -0.25 (95% CI -0.72 to 0.22) |
| IPAQ-E  (MET minutes) | 76 | 3673.6 (5205.5) | 57 | 4914.3 (7664.7) | -1240.66 (95% CI -968.70 to 3450.03) |
| General Self-efficacy | 89 | 32.2 (5.0) | 70 | 32.8 (4.3) | -0.79 (95% CI -1.98 to 0.40) |
| Confident manage pessary problems | 91 | 84.8 (18.0) | 69 | 71.1 (27.8) | 13.05 (95%CI 6.07 to 20.05) |
| Confident insert pessary | 92 | 95.8 (10.3) | 69 | 32.3 (33.8) | 59.64 (95%CI 52.16 -67.13) |
| Confident remove pessary | 92 | 96.5 (7.9) | 69 | 38.3 (35.4) | 51.65 (95%CI 44.00 -59.30) |
|  | N | n(%) | N | n(%) | Odds ratio |
| PGI-I, better | 92 | 41 (44.6%) | 69 | 15 (21.7%) | 4.10 (95%CI 1.98-8.47) |

PFDI-20, Pelvic Floor Distress Inventory-20; PISQ-IR, Prolapse Incontinence Sexual Questionnaire-IUGA Revised version; IPAQ-E, International Physical Activity Questionnaire for the Elderly; PGI-I: Patient Global Impression of Improvement

On treatment analysis. Model not repeated measures- dependent variable outcome at 4 years adjusted for baseline value and minimisation covariates with random effect of Centre.

**Supporting** **Table E: EQ-5D-5L health state utility scores at baseline, 6 months, 12 months, 18-months and 4-year follow-up, by randomised group**

| Assessment | Self-Management | Clinic-based Care | Self-Management | Clinic-based Care | p-value* |
| --- | --- | --- | --- | --- | --- |
|  | Mean (SD); n** | Mean (SD); n** | Median | Median |  |
| **Index score** |  |  |  |  |  |
| Baseline | 0.851 (0.170); 125 | 0.840 (0.185); 139 | 1.000 | 1.000 | 0.732 |
| 6 months | 0.841 (0.187); 125 | 0.829 (0.190); 139 | 0.814 | 0.814 | 0.593 |
| 12 months | 0.833 (0.193); 125 | 0.811 (0.192); 139 | 0.814 | 0.814 | 0.301 |
| 18 months | 0.823 (0.190); 125 | 0.819 (0.188); 139 | 0.814 | 0.814 | 0.856 |
| 4 year*** | 0.824 (0.214); 85 | 0.846 (0.170); 100 | 0.867 | 0.867 | 0.597 |
| **EQ-VAS** |  |  |  |  |  |
| Baseline | 83.28 (12.65); 125 | 82.40 (15.50); 139 | 85 | 85 | 0.912 |
| 6 months | 80.83 (14.61); 125 | 80.39 (15.84); 139 | 85 | 85 | 0.903 |
| 12 months | 79.59 (15.08); 125 | 79.50 (17.95); 139 | 80 | 80 | 0.524 |
| 18 months | 78.56 (17.35); 125 | 79.15 (16.80); 139 | 80 | 81 | 0.608 |
| 4 years*** | 78.94 (14.62); 81 | 78.71 (15.55); 99 | 80 | 83 | 0.830 |

* Mann–Whitney two sample test that data are from populations with the same distribution. A high p-value suggests that the two groups are very similar.

** Analysis sample.

***All available data are shown at 4-years

Note: Analyses by subgroup did not reveal statistically significant differences between SM and CBC groups (not shown-available upon request).

**Supporting** **Table F: Health care resource use in monetary terms by trial group over the 18-month follow-up and 4-year extension (intention-to-treat)**

|  | Self-management | | | | | Clinic-based Care | | | | |
| --- | --- | --- | --- | --- | --- | --- | --- | --- | --- | --- |
|  | N | Mean* (GBP£) | SD | Min | Max | N | Mean* (GBP£) | SD | Min | Max |
| Initial appointment** | 78 | 31.77 | 9.98 | 20.00 | 56.88 | 91 | 0 | 0 | - | - |
| Clinic visits Telephone support***  NHS Costs 18m**** |  | 475.17 | 473.45 | 0 | 2,132.84 |  | 779.72 | 705.86 | 31.20 | 3,663.52 |
| NHS costs 4y***** |  | 414.00 | 766.69 | 0 | 4,667.84 |  | 380.16 | 939.55 | 0 | 8,077.36 |
| Medications 18m |  | 15.52 | 45.57 | 0 | 348.00 |  | 24.90 | 79.88 | 0 | 667.88 |
| Medications 4y****** |  | 55.80 | 128.16 | 0 | 917.02 |  | 55.92 | 91.26 | 0 | 432.90 |
| *Mean calculations include zero reported resource use. All costs are deflated and discounted to the 2019/2020 prices so that costs are comparable across all time points.  **Training appointment that applies only to self-management group at beginning of trial (baseline).  ***From CRF data; some telephone appointments were costed as clinic visits if these were supposed to take place in person but were not due to COVID-19.  ****National health service and medication (also presented separately) costs at 18 months  ***** National health service costs between 18 months and 4 years. Recall period was up to 6 months prior to the 4-year point, reported resource use was deflated and discounted to 2019 prices. (p-value=0.861 the two groups are identical statistically speaking)  ******Medications used between 18 months and 4 years | | | | | | | | | | |

**Supporting** **Table G: Distribution of incremental costs and effects associated with self-management compared to clinic-based care over the 4 years of the TOPSY study (‘on treatment’)**

| Distribution of incremental costs and effects associated with self-management compared to clinic-based care over the 4 years of the TOPSY study (On treatment Pathway 1) | | | | | | | |
| --- | --- | --- | --- | --- | --- | --- | --- |
|  | N | Total Cost (£GBP) | Total QALYs | Incremental cost (Bootstrapped SE*) | Incremental QALYs (Bootstrapped SE*) | ICER | INB (Bootstrapped SE*) |
| Self-management | 84 | £ 756.85 | 3.245 | -479.43 (142.77) | 0.019  (0.073) | Dominated | £ 864.97 (£1,523.81) |
| Clinic-based care | 63 | £ 1,236.28 | 3.225 |  |  |  |  |
| Probability of cost-effectiveness at £20,000 WTP | | | | **71.32%** | | | |
| * Standard error based on 10,000 bootstrap resamples of incremental cost and effects  *Full completion implies that we only used data where participants have responded to all questions fully without any gaps at follow ups. Reason for dropping out was that some participants did not respond to questions at certain follow-ups. | | | | | | | |

**Supporting** **Figure A: PFIQ-7 predictive margins for SM and CBC over time**


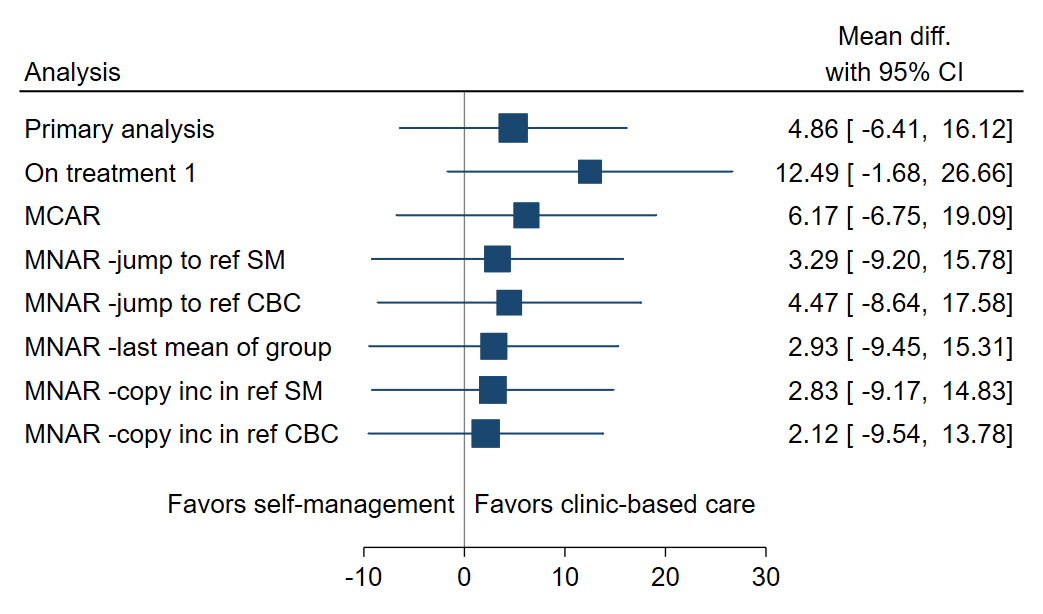


Sensitivity analysis based on Cro et al. 2018. In all scenarios the joint distribution of an individual’s observed and missing outcome data is multivariate normal with a mean vector from the individual’s allocated group up to the last observation time.

**MCAR**: Missing Completely At Randon.

**MNAR -Jump to ref** – Missing Not At Random, for missing observations the individual’s mean response profile follows that observed for stated reference group.

**MNAR Last mean carried forward.** Missing Not At Random, For missing observations, the individual’s means are set equal to the value of the marginal mean for her randomised treatment group at the last observed measurement.

**MNAR Copy increments in reference** Missing Not At Random, For missing observations the individual’s mean increments follow those for stated reference group.


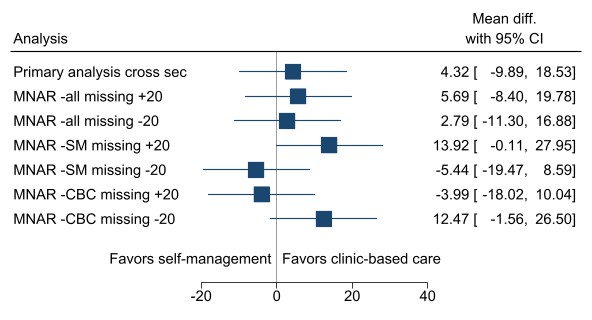


MNAR-all missing +20: Missing Not at Random assuming all participants (n=340) who had a missing PFIQ-7 score did worse, and their imputed score was increased by 20 points.

MNAR – all missing -20: Missing Not at Random assuming all all participants (n=340) who had a missing PFIQ-7 score did better, and their imputed score was decreased by 20 points.

MNAR-SM missing +20: Missing Not at Random assuming SM group only did worse and imputed score increased by 20 points

MNAR-SM missing -20: Missing Not at Random assuming SM group only did better and imputed score decreased by 20 points

MNAR–CBC missing +20:Missing Not at Random assuming CBC group only did worse and imputed score increased by 20 points

MNAR-CBC missing -20: Missing Not at Random assuming CBC group only did better and imputed score decreased by 20 points

The sensitivity analyses above are based on 4-year outcomes only, adjusted for baseline ie not using all longitudinal data. The primary analysis with 4-year data only is shown for comparison.

**Supporting** **Figure B: Sensitivity analysis**

**
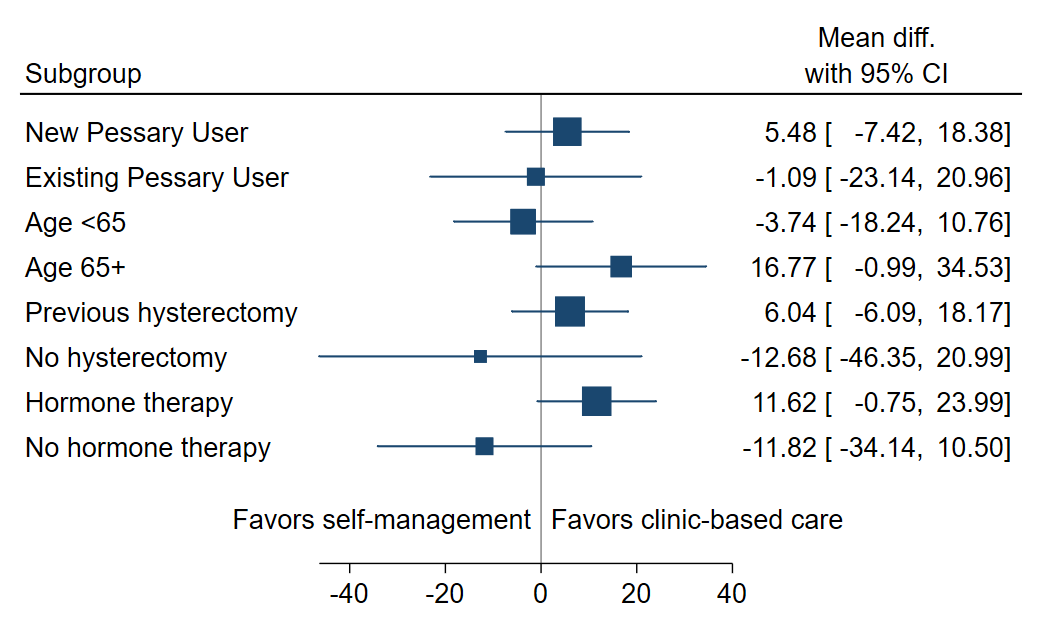
Supporting** **Figure C: Subgroup Analysis**


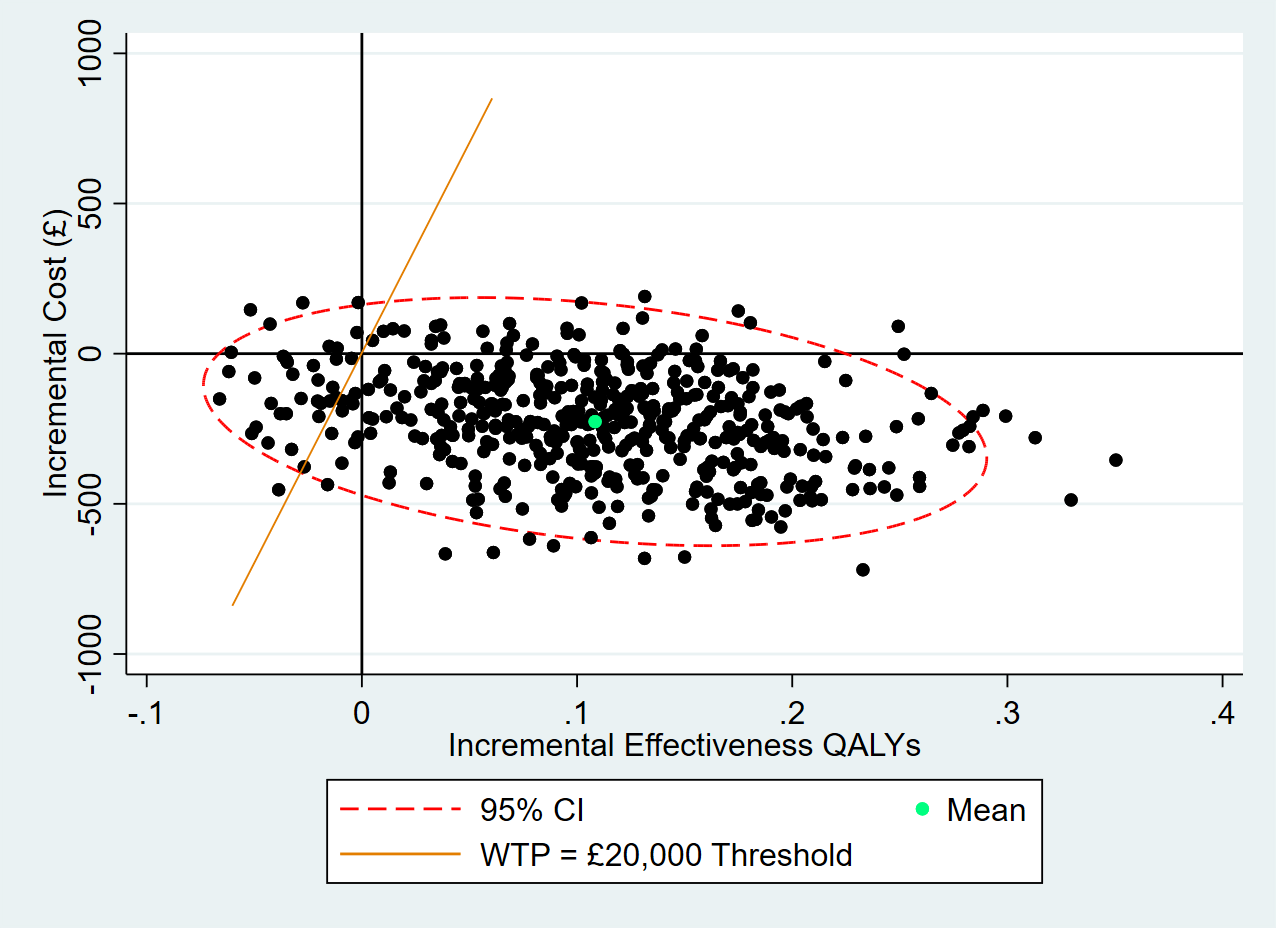


**Supporting** **Figure D: Incremental cost-effectiveness scatterplot**
